# Efficacy and aesthetic outcomes for quilting sutures in the prevention of seroma after mastectomy

**DOI:** 10.1038/s41598-023-29154-2

**Published:** 2023-02-02

**Authors:** Arthur Foulon, Albine Mancaux, Pierrick Theret, Philippe Naepels, Johanna Mychaluk, Philippe Merviel, Pascal Abboud, Raffaele Fauvet

**Affiliations:** 1 Service de Gynécologie Obstétrique, CHU Amiens Picardie, 1 Rond-Point Bd du Pr Christian Cabrol, 80000 Amiens, France; 2 Service de Gynécologie, CH Compiègne, 8 Avenue Henri Adnot, 60200 Compiègne, France; 3 Service de Gynécologie, CHU Brest, 2 Avenue Foch, 29200 Brest, France; 4 Université Occidentale de Bretagne, UFR de Médecine, 3 Rue Des Archives, 29238 Brest, France; 5 Service de Gynécologie, CH Soissons, 46 aAvenue du Général de Gaulle, 02200 Soissons, France; 6 Service de Gynécologie, CHU Caen Normandie, 1 Bd de La Côte de Nacre, 14000 Caen, France; 7 Université Caen Normandie, UFR de Médecine, Espl. de La Paix, 14000 Caen, France

**Keywords:** Breast cancer, Clinical trial design, Surgical oncology

## Abstract

Worldwide, mastectomy for breast cancer is one of the most frequently performed surgical procedures. As one of the main complications of mastectomy, seroma is associated with pain, infections and a prolonged hospital stay. We performed a prospective multicenter randomized trial to assess the efficacy and esthetic outcomes associated with quilting the skin flap. Eighty-seven patients were included. The proportion of patients with seroma on postoperative day 15 was significantly lower in the quilting group (12 out of 39 (30.8%)) than in a control group with conventional wound closure (21 out of 40 (52.5%); *P* = 0.05). The mean breast seroma volume was significantly lower in the quilting group (130.2 mL) than in the control group (236.8 mL; *P* = 0.02). There were no differences in the esthetic outcomes. The pain level on day 1 was similar in the quilting and control groups (mean visual analog scale score: 2.5 vs. 2.1, respectively; *P* = 0.3). Quilting the skin flap was associated with a lower prevalence of seroma and a lower seroma volume, and did not worsen the esthetic outcomes or pain levels. This technique is technically straightforward and should be offered to all patients scheduled for mastectomy.

## Introduction

In 2018, more than 2 million women worldwide were diagnosed with breast cancer^1^. The recent development of oncoplastic surgery has led to a fall in the number of mastectomies. Nevertheless, more than 20,000 mastectomies were performed in France in 2019^2^. Seroma (an accumulation of serous fluid under the skin flaps, due to mastectomy or axillary lymph node dissection) is one of the main complications of mastectomy and is even considered to be inevitable by some surgeons^3,4^. The incidence of seroma range from 15 to 90%^3^. This condition is associated with greater pain levels, a prolonged hospital stay, delayed wound healing, and infections (due to repeated drainage procedures) and may also delay the initiation of adjuvant treatments^3,5,6^. Lastly, seroma is also responsible for a higher cost of care^7^. Although the pathophysiology of seroma has not been characterized, the closure of dead spaces appears to be one of the best ways of preventing its occurrence^4,8^.

Flap fixations (to close dead space) consist in fixing the subcutaneous space to the pectoral fascia. Many suture- or fibrin-sealant-based techniques have been described for skin flap fixation^9^. To date, 15 prospective studies have evaluated the impact of skin flap fixation on seroma prevention^7,10–23^, and 8 of them were randomized^10,11,13,15,17–20^. Three of the five studies used conventional sutures, whereas Khater et al. used quilting with running sutures, and Granzier et al. combined sutures and tissue glue^11,13^. A recent review found that flap fixation reduces seroma formation and the need for seroma drainage after mastectomy but could not recommend a particular fixation technique^24^. Furthermore, one study evaluated the esthetic outcomes after skin flap fixation^13^. At present, there are no guidelines on flap fixation after mastectomy.

Hence, the objectives of the present study were to (i) compare the prevalence of seroma with or without quilting sutures 15 and 30 days after mastectomy, and (ii) evaluate the esthetic outcomes in the quilting and control groups.

## Patients and methods

We performed a prospective multicenter single-blind randomized controlled clinical trial. All patients due to undergo scheduled mastectomy with or without axillary lymph node dissection at Amiens Picardie University Hospital (Amiens, France), Compiegne General Hospital (Compiegne, France) or Soissons General Hospital (Soissons, France) were considered for enrollment. Patients were randomized into two groups: quilting suture or conventional wound closure. The study protocol was approved by the local institutional review board named *CPP Nord-Ouest II*, Amiens, France; reference: 2013-A00068-37. The study was registered at ClinicalTrials.gov, 09/09/2016 (cal tri). This work has been reported in line with Consolidated Standards of Reporting Trials (CONSORT) Guidelines. All methods were performed in accordance with the relevant guidelines and regulations.

### Inclusion and exclusion criteria

The main inclusion criteria were female sex, age 18 or over, and invasive or in situ breast cancer requiring mastectomy. The main non-inclusion criteria were pregnancy, immediate breast reconstruction, prophylactic mastectomy, and treatment with anticoagulants or antiplatelet agents. Patients unable to provide their written informed consent or to understand the study procedures were excluded.

### Procedure

All patients were provided with information about the study in a preoperative consultation and gave their written informed consent on the day before surgery. Included patients were randomized about 24 h before surgery. Randomization was stratified by center, body mass index, and whether or not axillary lymph node dissection was planned. Patients in the control group underwent conventional wound closure using subcutaneous stitches and intradermal sutures with absorbable monofilament to close the skin edges. Patients in the quilting group had the skin flap sutured to the pectoral fascia and then underwent conventional wound closure. The skin flap was fixed with absorbable polyfilament sutures. Two rows of continuous suturing were implemented for the upper and lower skin flaps. When the dead space was not sufficiently closed, one or two additional rows were implemented. Detailed instructions on closure techniques were given to all surgeons. After surgery, all patients underwent vacuum drainage in the mastectomy area and (if axillary lymph nodes had been dissected) in the axillary area. Seroma was then defined as the presence of serous fluid in the mastectomy area. Presence of a seroma was diagnosed by palpation but also according to the patient’s feeling (pain/feeling of swelling or tension). In case of doubt, ultrasound was performed. In the presence of a seroma, the volume was systematically quantified by a puncture. In each center, all operations were performed by experienced breast cancer surgeons. All surgeons used electrocautery for the skin flap dissection.

Esthetic outcomes were evaluated 90 days after surgery by the patient, the surgeons, and an third-party observer (a resident) on a Likert scale (“not at all satisfactory”, “somewhat satisfactory”, “satisfactory”, and “very satisfactory”). The residents evaluated the esthetic outcomes in a single-blind manner.

### Statistical analyses

The primary study endpoint was the presence of seroma 15 days after the surgery. Secondary endpoints were the operating time (in minutes), length of hospital stay (in days), the number of drainage procedures, the seroma volume drained in hospital on days 15, 30, and 90, postoperative pain (rated on a visual analog scale (VAS)), postoperative complications (delayed wound healing, infection, skin necrosis, lymphedema, and hematoma), and the esthetic outcomes.

In a descriptive analysis, normally distributed quantitative variables were quoted as the mean ± standard error of the mean (SEM). Groups were compared using a chi-squared test or Fisher’s exact test. Other variables were analyzed using a *t* test or Wilcoxson’s test. The threshold for statistical significance was set to *p* < 0.05.

### Ethics approval and consent to participate

The study protocol was approved by the local institutional review board (CPP Nord-Ouest II, Amiens, France; reference: 2013-A00068-37, February 21st, 2013). The study was registered at ClinicalTrials.gov (NCT02894021). This work has been reported in line with Consolidated Standards of Reporting Trials (CONSORT) Guidelines.

## Results

Between September 2013 and December 2017, 87 patients were included. Forty-three patients were randomly assigned to the quilting group and 44 were assigned to the control group. There were no intergroup differences with regard to age, body mass index, neoadjuvant therapy, or tumor histology (Table 1). The characteristics of the study population are summarized in Table 1.

**Table 1:** Population characteristics. IDC: infiltrative ductal carcinoma, ILC: infiltrative lobular carcinoma, DCIS: ductal carcinoma in situ, LCIS: lobular carcinoma in situ, ALND: axillary lymph node dissection, SLND: sentinel lymph node dissection, CT: chemotherapy.

	Quilting group (n = 43)	Control group (n = 44)	*p*
	*N* (%)	*N* (%)	
Characteristics			
Age	60.9 ± 15.89	57.8 ± 13	0.32
BMI ≤ 30 kg/m^2^	33 (76.7)	32 (72.7)	0.67
BMI > 30 kg/m^2^	10 (23.3)	12 (27.3)	
neoadjuvant CT	10 (23.3)	12 (27.3)	0.76
Tumor size (mm)	34.75 ± 26.64	37.86 ± 25.06	0.33
IDC	31 (72.1)	36 (81.8)	0.28
ILC	6 (14)	3 (6.8)	0.31
DCIS	13 (30.2)	11 (25)	0.59
Surgical procedure			
ALND	24 (55.8)	26 (59.1)	0.76
SLND	15 (34.9)	8 (18.2)	0.08

The operating time was 17 min longer in the quilting group than in the control group (mean: 128.7 ± 40.7 *vs.* 111.1 ± 41.2 min, *p* = 0.05). The total volume of seroma drained during the hospital stay was significantly lower in the quilting group (248.3 ± 207.2 ml) than in the control group (509.2 ± 46 ml; *p* = 0.008) (Table 2). The seroma volume drained from the breast area during the hospital stay was significantly lower in the quilting group (Table 2). The two groups did not differ significantly with regard to the volume of seroma drained from the axillary region (Table 2). The length of stay was significantly longer in the control group (Table 2). During the first 3 days after surgery, pain levels were similar in the quilting group and in the control group (Table 2). The peroperative and postoperative characteristics are summarized in Table 2.

**Table 2:** Peroperative and immediate postoperative characteristics.

	Quilting group (*N* = 43)	Control group (*N* = 44)	*p*
Operating time (min)	128.7 ± 40.7	111.1 ± 41.2	0.05
Breast seroma volume drained during hospital stay	130.2 ± 96.5	236.8 ± 203.60	0.02
Axillary seroma volume drained during hospital stay	118.1	272.4	0.2
Total seroma volume drained during hospital stay	248.3 ± 207.2	509.2 ± 469	0.01
Length of stay (days)	3.7 ± 1.4	4.8 ± 1.9	0.01
Time interval between surgery and breast drain removal (days)	2.5 ± 1	3.6 ± 1.9	0.01
Time interval between surgery and axillary drain removal (days)	3.1 ± 1.6	4.9 ± 1.9	0.001
Pain (visual analog scale)			
*Day 1*	2.5 ± 1.4	2.1 ± 1.1	0.33
*Day 2*	2.3 ± 1.5	2.4 ± 1.8	0.96
*Day 3*	2.2 ± 1.3	2 ± 1.7	0.35

Overall, 33 of the 87 patients (37.9%) had seroma 15 days after surgery (Table 3). At this time point, the proportion was significantly lower in the quilting group than in the control group [30.8% (12 out of 39) *vs.* 52.5% (21 out of 40), *p* = 0.05]. There were also significantly fewer patients who needed drainage at day 15 and day 30 after surgery (Table 3). The total drainage volume was significantly lower in the quilting group at day 15 (Table 3). Thirty days after surgery, the proportion of patients with seroma was 8.1% in the quilting group and 23.3% in the control group (Table 3); this difference was not statistically significant. There were no significant intergroup differences regarding pain levels, complications on days 15 and 30, and the time before initiation of adjuvant treatment (45.1 ± 20.9 days in the quilting group *vs.* 38.8 ± 15.2 in the control group; *p* = 0.2) (Table 3). The data on postoperative consultations are summarized in Table 3.

**Table 3:** Postoperative consultations.

	Quilting group	Control group	*p*
	*N* (%)	*N* (%)	
Consultation on day 15 (number of patients)	39	40	
seroma	12 (30.8)	21 (52.5)	0.05
drainage (number of patients)	7 (17.9)	18 (45)	0.015
drainage volume (ml)	76.4 ± 84.4	258 ± 165	0.015
Pain (VAS)	2.7 ± 1.4	3.8 ± 1.9	0.2
complications	6 (15.4)	6 (15)	0.96
*delayed wound healing*	0	0	
*infection*	1	1	
*skin necrosis*	1	4	
*lymphedema*	1	0	
*hematoma*	2	1	
*other*	3	1	
Consultation on day 30 (number of patients)	37	43	
seroma	3 (8.1)	10 (23.3)	0.07
drainage (number of patients)	1 (2.7)	7 (16.3)	0.04
drainage volume (ml)	130	105.7 ± 76.6	0.67
Pain (VAS)	2 ± 1.7	3.6 ± 1.6	0.14
complications	5 (13.5)	6 (14)	0.92
*delayed wound healing*	0	1	
*infection*	1	0	
*skin necrosis*	0	3	
*lymphedema*	1	0	
*hematoma*	1	2	
*other*	2	0	
Time interval between surgery and adjuvant therapy (days)	45.1 ± 20.9	38.8 ± 15.2	0.17

More than 80% of patients in padding group were satisfied with the esthetic outcome; this value was 76% in the control group (Table 4). Likewise, more than 80% of surgeons and residents were satisfied with the esthetic outcomes in both groups (Table 4). There were no significant differences between the two groups with regard to satisfaction with the esthetic outcomes (Table 4).

**Table 4:** Aesthetic results on postoperative day 90.

	Quilting group	Control group	*p*
Esthetic result	*N* (%)	*N* (%)	
Patient’s opinion	*N* = 22	*N* = 21	
*not at all satisfactory*	0	1 (4.8)	1
*somewhat satisfactory*	3 (13.6)	4 (19)	1
*satisfactory*	13 (44.8)	11 (52.4)	0.66
*very satisfactory*	6 (27.3)	5 (23.8)	0.79
Surgeon’s opinion	*N* = 21	*N* = 21	
*not at all satisfactory*	0	3 (14.3)	0.23
*somewhat satisfactory*	3 (14.3)	1 (4.8)	0.6
*satisfactory*	9 (42.8)	9 (42.9)	1
*very satisfactory*	9 (42.9)	8 (38)	0.75
Observer’s opinion	*N* = 20	*N* = 20	
*not at all satisfactory*	0	1 (5)	1
*somewhat satisfactory*	3 (15)	2 (10)	1
*satisfactory*	11 (55)	13 (65)	0.52
*very satisfactory*	6 (30)	4 (20)	0.47

## Discussion

Our results showed that quilting after mastectomy is associated with a significantly lower seroma prevalence 15 days after surgery (30.8% in the quilting group *vs.* 52.5% in the control group, *p* = 0.05).

Surgery still accounts for a large proportion of breast cancer treatments. For many years now, breast cancer surgeons have striven to find the best way of reducing seroma after breast surgery and (in some cases) axillary lymph node dissection or not. Firstly, closed vacuum drainage was developed to limit seroma. In a study published in 1993, patients with skin flap fixation had three times less seroma than other patients^25^. Kuroi et al. then affirmed that closing the dead space in the mastectomy area was one of the best ways of preventing seroma^4^. Seroma has many consequences: pain, prolonged hospital stay, delayed wound healing, infection (due to repeated aspirations), flap necrosis, delayed initiation of adjuvant treatments, repeat outpatient visits, and a higher cost of care—all of which can negatively influence the patient’s experience^3,5–7^.

Our results are in line with other studies of the same topic. Here, we showed that in the quilting group, seroma was significantly less prevalent on postoperative day 15 and the seroma drainage volume was significantly lower. In Sakkary et al.’s 2010 study, 40 patients scheduled for mastectomy were randomly assigned a flap fixation (interrupted suture) group or a conventional wound closure group. The patients in both groups underwent vacuum drainage. The seroma prevalence was significantly lower in the flap fixation group (10%) than in the control group (40%; *p* = 0.03)^10^. Mazouni et al.’s non-randomized study gave similar results for 82 patients undergoing mastectomy^12^. The investigators also closed the dead space with interrupted sutures, and the patients in both groups underwent vacuum drainage. The seroma prevalence was significantly lower in the skin flap fixation group (34.1%) than in the control group (58.5%; *p* = 0.03)^12^. In Mannu et al.’s study, 28% (15/54) patients in the quilting group developped seroma requiring aspiration *vs*. 69% (79/114) for patients without quilting after their mastectomy (*p* < 0.001)^14^. In this study, no patients in the quilting group had a drain^14^. Mean volume of seroma aspirated was although significantly lower in the quilting group (63 *vs.* 427 ml, *p* = 0.0008)^14^. Khater et al.’s study used quilting with running sutures^11^. Patients undergoing modified radical mastectomy were randomly assigned to one of two groups (with or without quilting of the skin flaps). Again, all patients underwent vacuum drainage. Seroma was significantly less frequent in the quilting group (20%) than in the control group (78.3%; *p* < 0.001)^11^. In their recent meta-analysis, Morarasu et al. observed that quilting sutures significantly reduced seroma formation (OR 0.32, 95% CI 0.21–0.49, *p* < 0.00001) and total volume of drainage (mean difference = 475 ml, 95% CI 337.58–612.40, *p* < 0.00001)^25^. In this study, quilting was not associated with complications such as surgical site infections, hematoma or flap necrosis^25^.

Quilting also reduces the number of seroma drainage procedures. According to the literature, reducing seroma incidence and seroma volume decreases the risk of surgical site infections—although we did not find that in the present study^26^. Lower frequencies of seroma drainage procedures and skin infections are likely to be associated with greater levels of patient satisfaction.

The seroma volume drained from mastectomy area during the hospital stay was significantly lower in the quilting group because the duration of drainage was shorter. Moreover, the proportion of patients requiring seroma drainage was also significantly lower in the quilting group at day 15 and at day 30. In Khater et al.’s study, the total drainage volume was also significantly lower for patients in the quilting group^11^. These results confirmed that it is not necessary to maintain drainage in the mastectomy area for a long time to observe the efficacy of quilting.

Fixing the skin flap by quilting typically takes longer than fixing it with separate sutures. Indeed, the operating time was significantly longer in the quilting group than in the control group in our study (mean: 128.7 min) and in the study by Khater et al. (mean: 127 min)^11^. In contrast, the operating times in the two groups (separate sutures vs. a control group) did not differ in Mazouni et al.’s study^12^.

Our prospective trial has evidenced a significant reduction in the length of stay in the skin flap fixation group (3.7 days, vs. 4.8 days in the control group; *p* = 0.009). In a retrospective study, the length of stay was also significantly lower for patients that underwent skin flap fixation by quilting after mastectomy^27^. These results are confirmed by the meta-analysis of Morarasu et al., they found a significant mean hospitalization of 0.7 days in favor of quilting suture (*p* = 0.007)^25^. This is a major advantage for patients and is associated with lower levels of stress, fewer nosocomial infections, and a faster return to baseline physical and psychological states^28^. Moreover, the time to initiation of adjuvant treatments (e.g. chemotherapy) was not significantly longer in our quilting group–constituting an additional argument in favor of this technique. In fact, quilting the skin flap did not lead to more discomfort or more complications and so did not delay adjuvant treatments. This is important advantage because delayed access to adjuvant treatment can impact the patient’s survival^29^.

Ten Wolde et al. found that postoperative pain was a limitation of skin fixation^30^. We did not observe a significant difference in post-operative pain levels between our two study groups, as was also the case in Mazouni et al.’s study^12^. However, the precise relationship between the skin flap fixation technique and postoperative pain has not been investigated. Quilting the skin flap to the pectoral muscle can induce esthetic sequelae, such as skin dimpling. Few studies have evaluated esthetic outcomes. In Granzier et al.’s recent study, patients were randomized into three groups: conventional wound closure, skin flap fixation with suture, or skin flap fixation with tissue glue^13^. The patients evaluated their esthetic outcomes at 3 months and one year, and there were no significant differences between the groups. This was also the case in our study; there were no intergroup differences in the esthetic outcomes—regardless of whether the latter were judged by the patient, the surgeon, or a resident. To the best of our knowledge, our study is the first to have included an evaluation of esthetic outcomes by surgeons and other observers. Quilting the skin flap with sutures is beneficial for patients with regard to seroma prevalence and volume, and is not associated with greater pain levels or worse esthetic outcomes.

A number of other skin flap fixation techniques have been developed. Some investigators have studied the use of TissuGlu^®^^31,32^. To date, this technique has not been linked to a lower prevalence of seroma in mastectomy area—although its utility after axillary lymph node dissection has been proven^33^.

## Conclusion

Quilting the skin flap was associated with a lower seroma incidence, a lower number of seroma drainage procedures, and a lower seroma drainage volume in the mastectomy area. This provides the patients with several benefits: a shorter hospital stay and less discomfort. Furthermore, quilting the skin flap does not worsen the esthetic outcomes or postoperative pain levels and does not delay the initiation of adjuvant treatments. This technique is technically straightforward and should be offered to all patients scheduled for mastectomy.

## Data Availability

The datasets used and/or analysed during the current study available from the corresponding author on reasonable request. All data will be available, accessible, discoverable and usable.
